# Suppression of choroidal neovascularization by silencing of long non-coding RNA IPW

**DOI:** 10.18632/aging.202822

**Published:** 2021-04-04

**Authors:** Tian-Jing Yang, Mu-Di Yao, Ya-Nan Sun, Xiu-Miao Li, Qin Jiang, Biao Yan

**Affiliations:** 1 The Fourth School of Clinical Medicine, Nanjing Medical University, Nanjing, China; 2 The Affiliated Eye Hospital, Nanjing Medical University, Nanjing, China; 3 Eye Institute, Eye & ENT Hospital, Shanghai Medical College, Fudan University, Shanghai, China; 4 NHC Key Laboratory of Myopia Fudan University, Key Laboratory of Myopia, Chinese Academy of Medical Sciences, Shanghai, China; 5 Shanghai Key Laboratory of Visual Impairment and Restoration, Fudan University, Shanghai, China

**Keywords:** long noncoding RNA, choroidal neovascularization, choroidal sprouting, miRNA

## Abstract

Long noncoding RNAs (lncRNAs) have emerged as the key regulators in the pathogenesis of human disorders. This study aimed to investigate the role of lncRNA-IPW in the progression of choroidal neovascularization (CNV) and the underlying molecular mechanism. IPW was significantly up-regulated in the choroidal tissues of laser-induced CNV mice and in the endothelial cells in response to hypoxic stress. IPW silencing led to reduced formation of CNV in laser-induced CNV model and ex vivo choroidal sprouting model, which could achieve similar therapeutic effects of anti-VEGF on CNV formation. Silencing or transgenic overexpression of IPW could alter endothelial cell viability, proliferation, migration, and tube formation ability *in vitro*. Mechanistically, IPW silencing led to increased expression of miR-370. Increased miR-370 could mimic the effects of IPW silencing on CNV formation and endothelial angiogenic phenotypes *in vivo* and *in vitro*. This study suggests that IPW silencing is a promising strategy for the treatment of neovascular ocular diseases.

## INTRODUCTION

Age-related macular degeneration (AMD) is the major cause of irreversible visual impairment and central vision loss among elderly people [1, 2], which results from the development of choroidal neovascularization (CNV) associated with overlying retinal damage. New vessels from the choroid invade the subretinal space through Bruch’s membrane, inducing the formation of fibrovascular tissues. Immature blood vessels may lead to fluid leakage, retinal hemorrhage, retinal pigment epithelial detachment, and subretinal fibrosis. The pathophysiology of AMD is complex and multifactorial. Genetic predisposition, metabolic dysfunction, inflammatory processes, choroidal ischemia, and hypoxic damage have been implicated in the pathogenesis of CNV [3–5]. Current therapy for CNV formation is mainly through the inhibition of vascular endothelial growth factor (VEGF) [6, 7]. However, the recurrence of CNV and frequent anti-VEGF drug administration may pose serious social and economic burden [8]. Thus, it is required to further understand the mechanism of CNV.

The pathogenesis of CNV is associated with genetic factors and environmental factors. The genetic factors include common and rare genetic, copy number and mitochondrial sequence variations, and epigenetics [9]. The environmental factors include smoking, obesity, and dietary factors [10]. Epigenetic mechanisms can alter gene expression through regulating DNA methylation, histone modification, chromatin remodeling, and non-coding RNAs [11].

Long non-coding RNAs are a diverse group of regulatory non-coding RNA species longer than 200 nt [12, 13]. They are expressed in cell or development specific pattern and engage in many biological processes across every branch of life, such as transcriptional regulation, nuclear domains organization, and proteins or RNA molecules regulation [14, 15]. Aberrant expression of lncRNAs has been observed in many diseases, such as cancer, cardiac, neurological, and metabolic diseases [16–19]. However, the role of lncRNAs in choroidal neovascularization remains largely unknown.

IPW is a long non-coding RNA, which is identified in, an epigenetic disorder, Prader-Willi syndrome (PWS) [20]. IPW is located in the critical region of the PWS locus and is an important regulator of the DLK1-DIO3 region [21]. Disruption of the IPW region is associated with neurogenic disorders in humans. IPW overexpression can lead to the downregulation of maternally expressed genes (MEGs) in the imprinted DLK1-DIO3 locus on chromosome 14, suggesting a potential role in epigenetic regulation [21]. Since the pathogenesis of AMD is potentially regulated by epigenetic mechanism, we thus investigated the role of IPW in CNV and the underlying molecular mechanism.

In this study, we revealed that IPW expression was significantly up-regulated in the laser-induced CNV lesions and in the endothelial cells in response to hypoxic stress. IPW silencing significantly reduced the formation of CNV in laser-induced CNV model and choroidal sprouting model. IPW regulated CNV development via regulating miR-370 level. This study underscores the importance of IPW in maintaining choroidal vascular stability. Silencing of IPW may provide a promising strategy for the treatment of neovascular ocular diseases.

## RESULTS

### LncRNA-IPW is significantly up-regulated in laser-induced CNV lesions and in endothelial cells upon hypoxic stress

**Figure 1:**
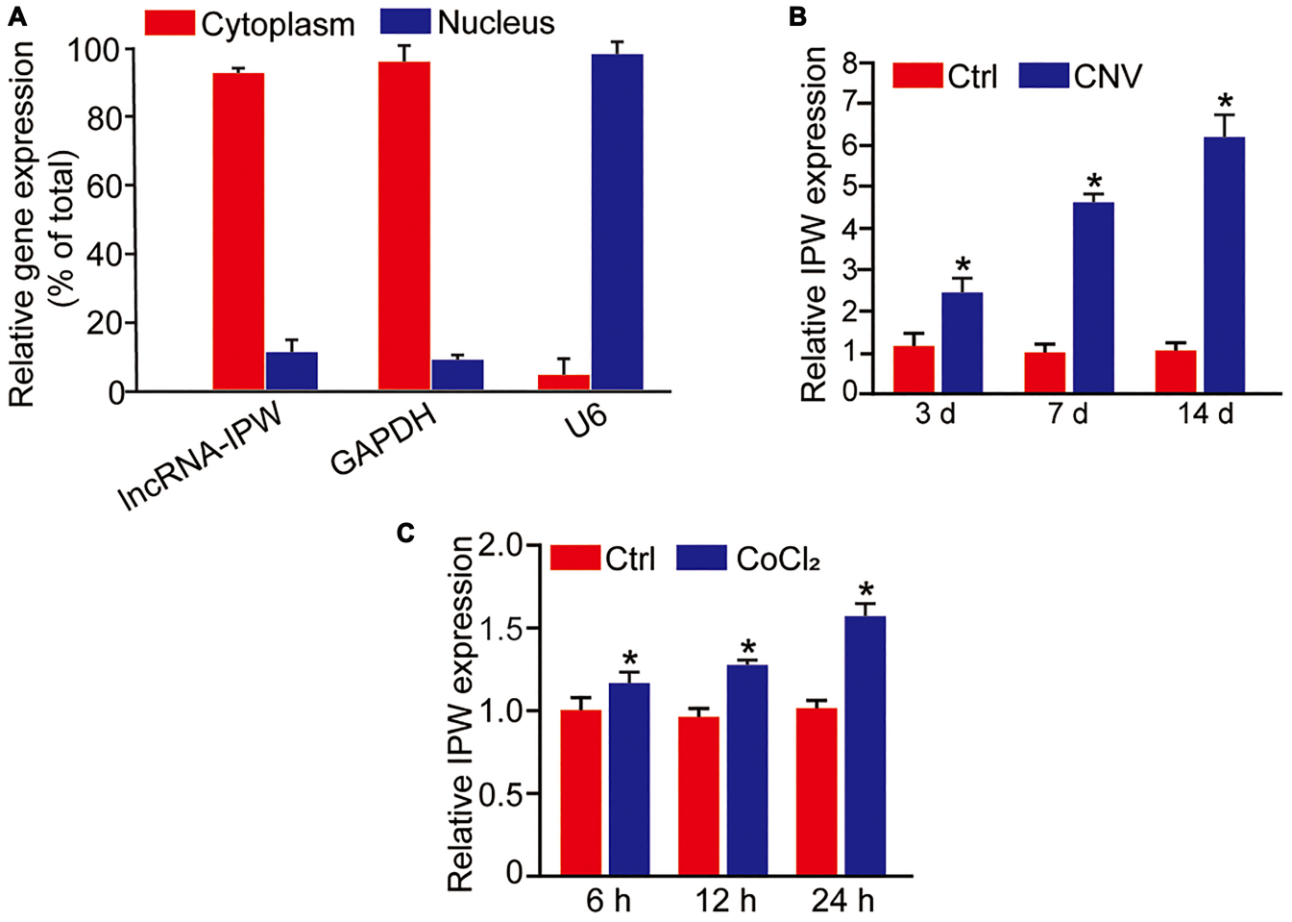
**LncRNA-IPW is significantly up-regulated in laser-induced CNV lesions and endothelial cells upon hypoxic stress.** (**A**) The expression of nucleus control transcript (U6), cytoplasm control transcript (GAPDH), and IPW was detected by qRT-PCRs in the nucleus and cytoplasm fractions of RF/6A cells (*n* = 4). (**B**) qRT-PCRs were performed to detect the expression of IPW in the RPE/choroid complex of C57BL/6J mice on day 3, day 7, and day 14 after laser photocoagulation (*n* = 5 animal per group; ^*^P < 0.05; Mann-Whitney *U* test). (**C**) RF/6A cells were exposed to 200 μM CoCl_2_ for the indicated time points. qRT-PCRs were performed to detect the expression of IPW (*n* = 4; ^*^P < 0.05; Student’s *t*-test).

We first determined the expression pattern of IPW in chorioretinal endothelial cells (RF/6A). qRT-PCR assays revealed that IPW was mainly expressed in the cytoplasm of RF/6A cells (Figure 1A). We then determined whether IPW was dysregulated in the CNV model. qRT-PCR assays showed that IPW was significantly up-regulated in the RPE/choroid complex on day 3, day 7, and day 14 after laser photocoagulation (Figure 1B). Hypoxia is considered as a critical factor in the etiology of CNV [22]. RF/6A cells were exposed to CoCl_2_ for 6 h, 12 h, and 24 h to mimic hypoxia stress. qRT-PCR assay revealed that IPW was significantly up-regulated in hypoxic group in a time-dependent manner (Figure 1C).

### Silencing of lncRNA-IPW reduces endothelial angiogenic function *in vitro*

**Figure 2:**
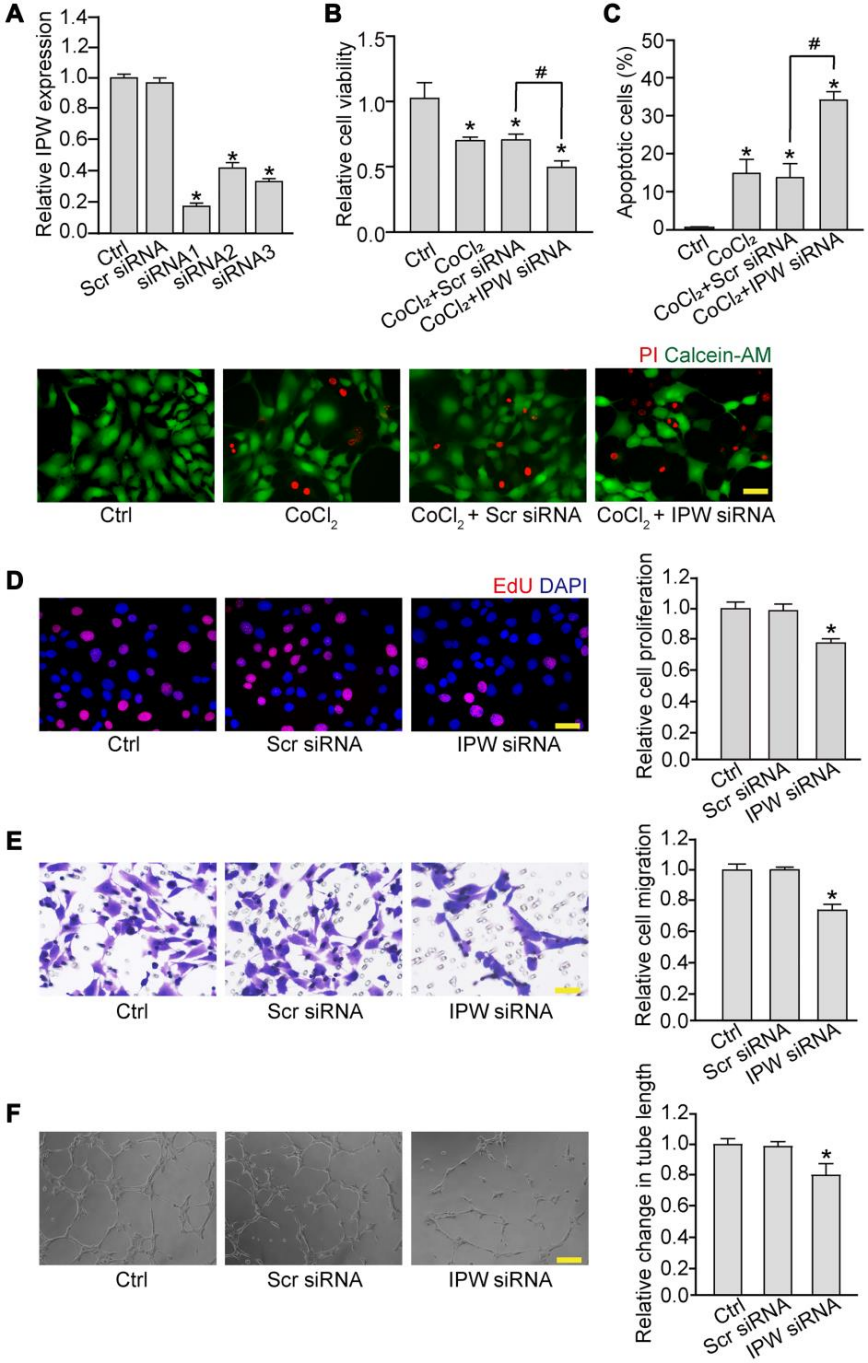
**Silencing of lncRNA-IPW reduces endothelial angiogenic function *in vitro*.** (**A**) RF/6A cells were transfected with scrambled (Scr) siRNA, IPW siRNA, or left untreated (Ctrl) for 24 h. qRT-PCRs were performed to detect IPW expression (*n* = 4; ^*^P < 0.05 versus Ctrl group; One-way ANOVA). (**B** and **C**) RF/6A cells were transfected with Scr siRNA, IPW siRNA, or left untreated for 24 h, and then exposed with CoCl_2_ (200 μmol/L) to mimic hypoxic stress for 24 h. The group without CoCl_2_ treatment was taken as the Ctrl group. Cell viability was detected by MTT assays (B; *n* = 4; ^*^P < 0.05 versus Ctrl group; One-way ANOVA). Apoptotic cells were detected by PI/Calcein-AM staining. Green: live cells; red: dead or dying cells. Scale bar: 50 μm (C; *n* = 4; ^*^P < 0.05 versus Ctrl group; ^#^*P* < 0.05 CoCl_2_ + IPW siRNA versus CoCl_2_ + Scr siRNA; One-way ANOVA). (**D**–**F**) RF/6A cells were transfected with Scr siRNA, IPW siRNA, or left untreated (Ctrl) for 24 h. Cell proliferation was determined by EdU incorporation assay. Blue: DAPI; red: EdU. Scale bar: 20 μm (D; *n* = 4; ^*^P < 0.05 versus Ctrl group; One-way ANOVA). The migration of RF/6A cells was measured using Transwell assays. These cells migrated through the chamber were quantified. Scale bar: 50 μm (E, *n* = 4; ^*^P < 0.05 versus Ctrl group; One-way ANOVA). The tube-like structures were observed 6 h after cell seeding on the matrix. The average length of tube formation for each field was statistically analyzed. Scale bar: 200 μm (F, *n* = 4; ^*^P < 0.05 versus Ctrl group; One-way ANOVA).

We then determined whether IPW regulated endothelial angiogenic function *in vitro*. Three siRNA sequences were designed to reduce the expression of IPW. The silencing efficiency was determined by qRT-PCR assays. All siRNAs could reduce the expression of IPW in RF/6A cells. IPW siRNA1 was selected for subsequent study because it had the greatest silencing efficiency (Figure 2A). By contrast, IPW overexpression led to increased expression of IPW compared with the control group in RF/6A cells (Supplementary Figure 1). MTT assays showed that IPW silencing significantly decreased the viability of RF/6A cells under hypoxic condition (Figure 2B). PI/Calcein-AM staining revealed that IPW silencing accelerated hypoxia-induced endothelial cell apoptosis as shown by increased number of PI-positive cells (Figure 2C). EdU incorporation assay showed that IPW silencing reduced the proliferation ability of RF/6A cells (Figure 2D). Transwell migration assays showed that IPW silencing decreased the migratory ability of RF/6A cells (Figure 2E). Tube formation assay showed that the relative tube length was significantly reduced in IPW silencing group compared with the control group (Figure 2F). By contrast, IPW overexpression could lead to increased cell viability, reduced endothelial cell apoptosis, increased proliferative ability, migration ability, and tube formation ability (Supplementary Figure 1). Collectively, these results suggest that IPW is a crucial regulator of endothelial angiogenic function.

### Silencing of lncRNA-IPW inhibits experimental choroidal neovascularization *in vivo*

**Figure 3:**
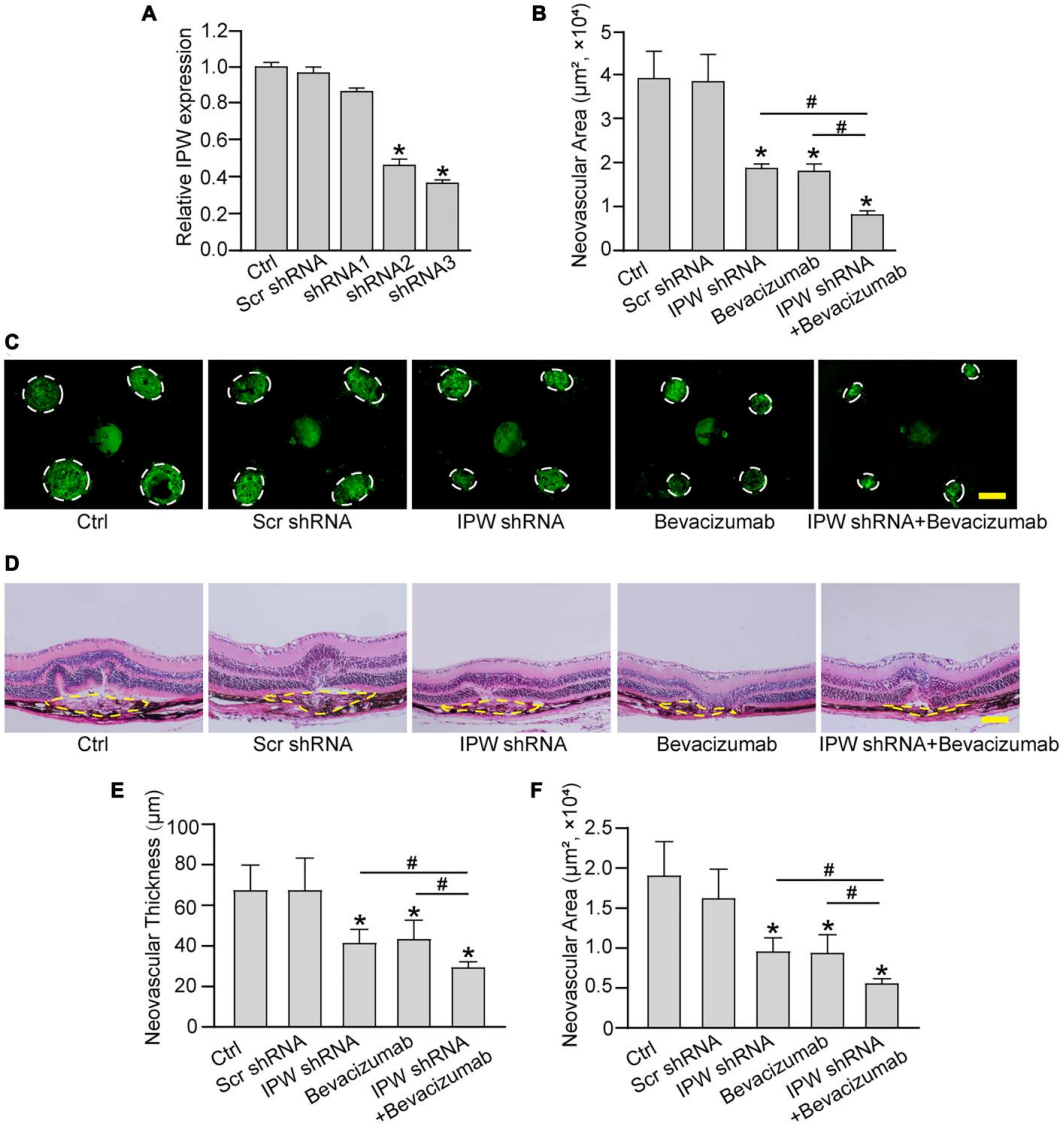
**Silencing of lncRNA-IPW inhibits experimental choroidal neovascularization *in vivo*.** (**A**) C57BL/6 mice received an intravitreal injection of scrambled (Scr) shRNA, IPW shRNA, or left untreated (Ctrl). qRT-PCRs were conducted to detect IPW expression at day 14 after intravitreal injection (*n* = 6 animals/group; Kruskal-Wallis test). (**B**–**F**) C57BL/6 mice received an intravitreal injection of Scr shRNA, IPW shRNA3, bevacizumab, IPW shRNA3 plus bevacizumab, or left untreated (Ctrl). After 14 days, the mice were euthanized and the RPE/choroid complexes were dissected and flat-mounted. The blood vessels were stained by Isolectin-B4 and neovascular area was calculated (B; *n* = 6 animals/group; Kruskal-Wallis test). (C) The representative images of Isolectin-B4 staining were shown on day 14 after laser photocoagulation. Green staining indicated the CNV lesion. Dashed lines delineate the lesions. Scale bar: 200 μm (C). (D–F) Histological sections of HE stained retinal sections from mice on day 14 after laser photocoagulation. Typical sections of laser-burned eye stained with HE, with the lesion delineated by the dashed line (D). Neovascular degree was estimated by neovascular thickness (E; *n* = 6 animals/group; Kruskal-Wallis test) and neovascular area (F; *n* = 6 animals/group; Kruskal-Wallis test). Scale bar: 100 μm. ^*^P < 0.05 versus Ctrl group; ^#^*P* < 0.05 versus IPW shRNA plus bevacizumab.

To determine the role of IPW in the formation of choroidal neovascularization, we designed 3 different short hairpin RNA (shRNAs) for IPW silencing. Two shRNAs could significantly reduce IPW expression. We selected IPW shRNA3 in the following study because it had the greatest silencing efficiency (Figure 3A). The area of CNV lesion was measured on day 14 after CNV formation. IPW silencing could reduce the formation of CNV by 54% compared with the CNV group, showing a similar effect as anti-VEGF drug (Figure 3B and 3C). The histology of CNV lesion at the same time point was further characterized. In the HE-stained paraffin section, the reduction of neovascular area and neovascular thickness was observed in the samples from IPW shRNA-injected group (Figure 3D). Moreover, intravitreal treatment of bevacizumab plus IPW shRNA showed a better outcome as shown by decreased neovascular thickness (Figure 3E) and neovascular area (Figure 3F). By contrast, IPW overexpression accelerated CNV formation compared with Ctrl group (Supplementary Figure 2). Collectively, these results indicate that IPW is involved in the formation of CNV *in vivo*.

### LncRNA-IPW regulates choroidal sprouting in *ex vivo* explant model

**Figure 4:**
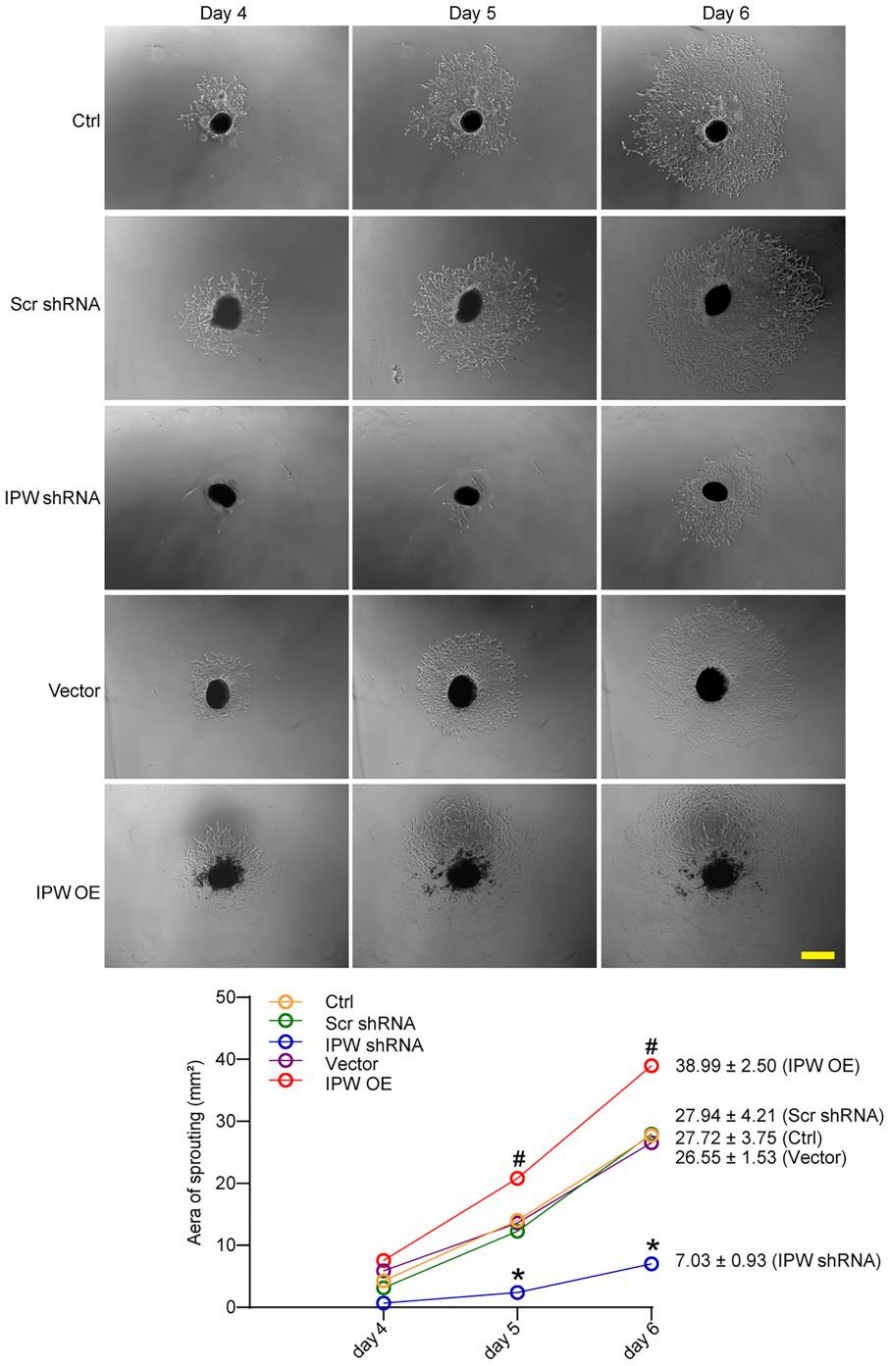
**LncRNA-IPW regulates choroidal sprouting in *ex vivo* explant model.** C57BL/6 mice received an intravitreal injection of Scr shRNA, IPW shRNA, vehicle (Vector), IPW overexpression vector (IPW OE), or left untreated (Ctrl, A-C). On day 14, RPE/choroid complexes were dissected. The peripheral regions of RPE complexes were cut into 1 mm × 1 mm pieces and seeded. The sprouting potency of choroidal explants were photographed on day 4, day 5, and day 6 (*n* = 6; Kruskal-Wallis test). The representative images of choroidal sprouting were shown. Meanwhile, the quantification results of sprouting area were shown. Scale bar: 1 mm.^*^P < 0.05 IPW shRNA versus Ctrl group; ^#^*P* < 0.05 IPW OE versus Ctrl group.

Next, we determined the role of IPW in choroid sprouting using the *ex vivo* CNV model. The choroid was dissected and embedded in Matrigel and cultured for 7 days. The area occupied by the migrated cells was quantified. The result showed that the sprouting area from the explant was significantly reduced after IPW silencing (Figure 4). By contrast, IPW overexpression led to increased sprouting ability compared with the vector group (Figure 4).

### LncRNA-IPW regulates the expression of genes located in DLK1-DIO3 locus

**Figure 5:**
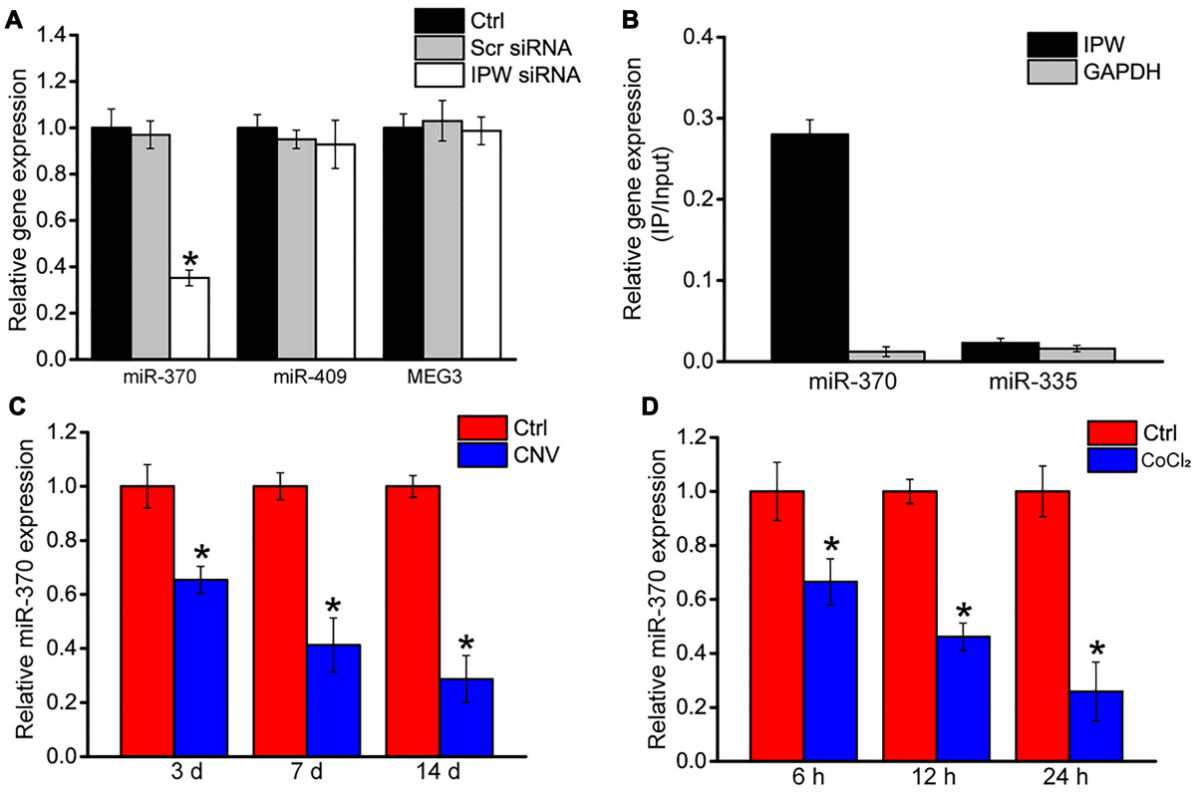
**LncRNA-IPW regulates the expression of genes located in DLK1-DIO3 locus.** (**A**) RF/6A cells were transfected with scrambled (Scr) siRNA, lncRNA-IPW siRNA, or left untreated (Ctrl) for 24 h. qRT-PCRs were performed to detect the expression of miR-370, miR-408, or MEG3 (*n* = 4; ^*^P < 0.05; One-way ANOVA). (**B**) The 3’-end biotinylated miRNA duplexes were transfected into RF/6A cells. After streptavidin capture, the levels of IPW and GAPDH in the input and bound fractions were detected using qRT-PCRs. The relative immunoprecipitate (IP)/input ratios were plotted. (**C**) qRT-PCRs were performed to detect the expression of miR-370 in the RPE/choroid complex of C57BL/6J mice on day 3, day 7, and day 14 after laser photocoagulation (*n* = 6 animal per group; ^*^P < 0.05; Mann-Whitney *U* test). (**D**) RF/6A cells were exposed to 200 μM CoCl_2_ for the indicated time. qRT-PCRs were performed to detect the expression of miR-370 (*n* = 4; ^*^P < 0.05; Student’s *t*-test).

Previous studies have reported that IPW regulates the expression of genes located in the imprinted DLK1-DIO3 locus, such as miR-370, miR-409, and MEG3 [23]. In RF/6A cells, we showed that silencing of IPW led to reduced expression of miR-370 but had no effect on the expression of miR-409 or MEG3 (Figure 5A). Using the biotin-coupled miR-370, we observed greater enrichment of IPW in miR-370-captured fraction compared to the negative control, biotinylated miR-335 (Figure 5B). The level of miR-370 expression was significantly reduced in laser-induced CNV lesions and endothelial cells upon hypoxic stress, displaying an opposite expression trend compared with IPW expression (Figure 5C and 5D).

### LncRNA-IPW/miR-370 interaction regulates endothelial angiogenic function *in vitro*

**Figure 6:**
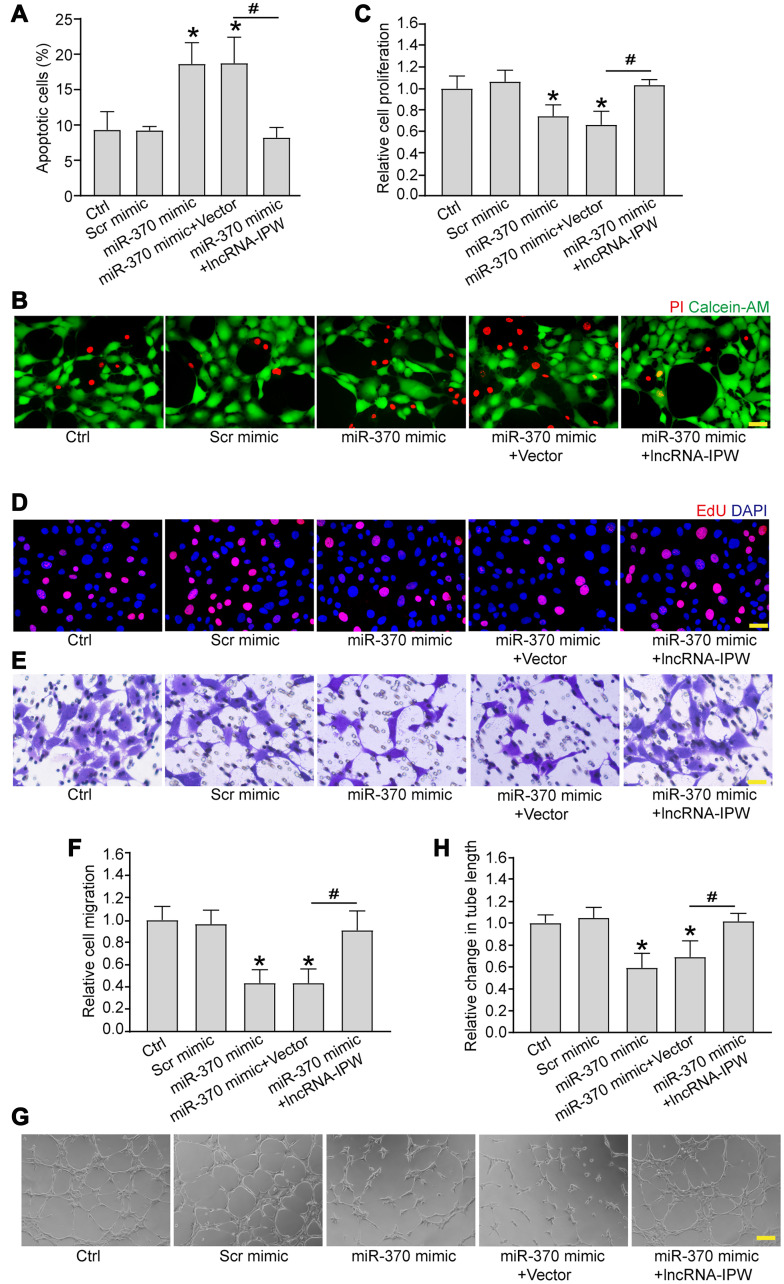
**LncRNA-IPW/miR-370 interaction regulates endothelial angiogenic function *in vitro*.** (**A** and **B**) RF/6A cells were transfected with scrambled (Scr) mimic, miR-370 mimic, miR-370 mimic plus pcDNA3.0 vector (Vector), miR-370 mimic plus pcDNA3.0-IPW, or left untreated (Ctrl) for 24 h, and then exposed with CoCl_2_ (200 μmol/L) to mimic hypoxic stress for 24 h. Apoptotic cells were determined by PI/Calcein-AM staining and quantified. Green: live cells; red: dead or dying cells. Scale bar: 50 μm (*n* = 4; ^*^P < 0.05 versus Ctrl group; ^#^*P* < 0.05 miR-370+IPW versus miR-370+vector; One-way ANOVA). (**C**–**H**) RF/6A cells were transfected with Scr mimic, miR-370 mimic, miR-370 mimic plus pcDNA3.0 vector, miR-370 mimic plus pcDNA3.0-IPW, or left untreated (Ctrl) for 24 h. Cell proliferation was detected by EdU staining and quantified. DAPI, blue; EdU, red. Scale bar: 20 μm (C and D; *n* = 4; ^*^P < 0.05 versus Ctrl group; ^#^*P* < 0.05 miR-370 + IPW versus miR-370+vector; One-way ANOVA). The migration of RF/6A cells was detected using Transwell assay and quantified. Scale bar: 100 μm (E and F; *n* = 4; ^*^P < 0.05 versus Ctrl group; ^#^*P* < 0.05 miR-370 + IPW versus miR-370 + vector; One-way ANOVA). The tube-like structures were observed at 6 h after cell seeding on the matrix. The average length of tube formation for each field was statistically analyzed. Scale bar: 200 μm (G and H; *n* = 4; ^*^P < 0.05 versus Ctrl group; ^#^*P* < 0.05 miR-370 + IPW versus miR-370 + vector; One-way ANOVA).

We next determined whether miR-370 was involved in the regulation of endothelial angiogenic function. Transfection of miR-370 mimic led to increased apoptosis in response to hypoxic stress (Figure 6A and 6B). Increased miR-370 led to reduced cell proliferation (Figure 6C and 6D), reduced cell migration (Figure 6E and 6F), and reduced tube formation activity compared to Ctrl group (Figure 6G and 6H). Moreover, IPW overexpression could rescue the inhibitory effects of miR-370 mimic on endothelial angiogenic function (Figure 6A–6H).

Next, we employed TargetScan to predict the potential target genes of miR-370. Two candidate genes, including KDR and BMP, aroused our interest due to their roles in the maintenance of vascular homeostasis. miR-370 mimic transfection significantly down-regulated KDR and BMP expression (Supplementary Figure 3A). The 3’-UTR of KDR and BMP gene was cloned into the luciferase vector and co-transfected with miR-370 mimic into RF/6A cells. A significant reduction in luciferase activity was detected in the presence of miR-370 mimic, whereas the mutation of miR-370 target site completely abolished this repression (Supplementary Figure 3B). Moreover, IPW silencing reduced the expression level of KDR and BMP *in vitro* (Supplementary Figure 3C).

### LncRNA-IPW/miR-370 interaction regulates the development of CNV *in vivo* and *ex vivo*

**Figure 7:**
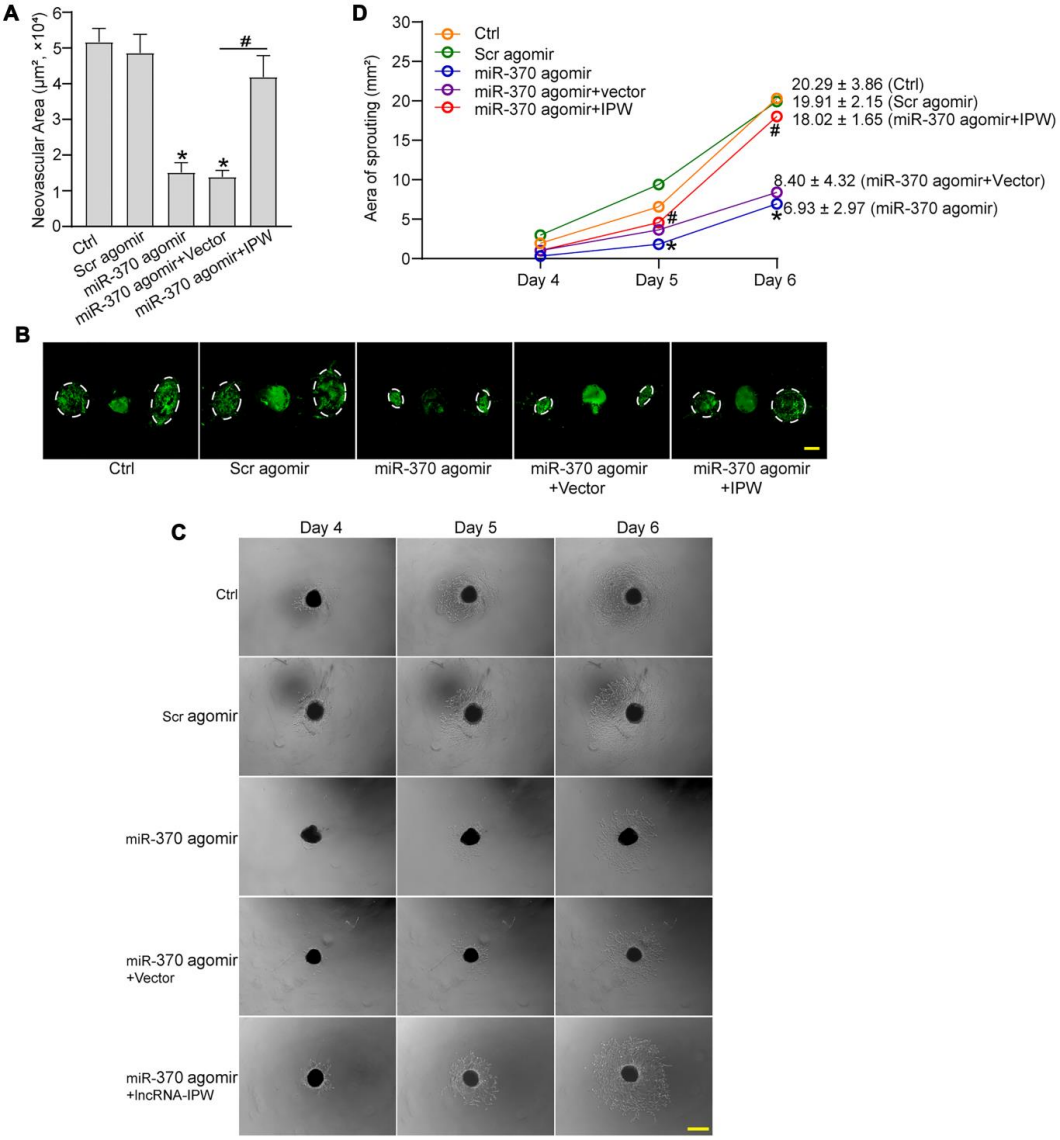
**LncRNA-IPW/miR-370 interaction regulates the development of CNV *in vivo* and *ex vivo*.** (**A** and **B**) The mice were injected with scrambled (Scr) agomir, miR-370 agomir, miR-370 agomir plus AAV vector, miR-370 agomir plus AAV-IPW, or left untreated (Ctrl). On day 14 after laser photocoagulation, the mice were euthanized and RPE/choroid complexes were dissected and flat-mounted. The blood vessels were stained with Isolectin-B4 (*n* = 6 animals/group; ^*^P < 0.05 versus Ctrl group; ^#^*P* < 0.05 miR-370 agomir group versus miR-370 agomir plus IPW group; Kruskal-Wallis test). The representative images of Isolectin-B4-stained flat-mounted choroid on day 14 after laser induction. Dashed lines delineate lesion areas. Scale bar: 200 μm (B). (**C** and **D**) The mice received an intravitreal injection of Scr agomir, miR-370 agomir, miR-370 agomir plus Vector, miR-370 agomir plus IPW, or left untreated (Ctrl). On day 14 after laser photocoagulation, the RPE/choroid complexes were dissected and choroidal explants were performed as previously described. The representative images of choroidal explants after 4-day, 5-day, and 6-day culture were shown (C). Scale bar: 500 μm. Meanwhile, the quantification results of sprouting areas were shown (D; *n* = 6 animals/group; ^*^P < 0.05 miR-370 agomir versus Ctrl group; ^#^*P* < 0.05 miR-370 agomir + IPW OE versus miR-370 agomir; Kruskal-Wallis test). Scale bar: 1 mm.

We next determined the role of miR-370 in CNV formation *in vivo* and *ex vivo*. In laser-induced CNV model, miR-370 agomir inhibited the formation of CNV compared with the CNV group. By contrast, IPW overexpression rescued the inhibitory effects of miR-370 agomir on CNV formation (Figure 7A and 7B). In the *ex vivo* choroidal sprouting assay, miR-370 agomir led to reduced sprouting capacity of choroidal explants compared with Ctrl group. By contrast, IPW overexpression abolished the inhibitory effects of miR-370 agomir on CNV formation and led to increased sprouting capacity of choroidal explants (Figure 7C and 7D). Collectively, the above-mentioned results indicate that IPW/miR-370 interaction is involved in the regulation of CNV formation *in vivo* and *ex vivo*.

## DISCUSSION

Age-related macular degeneration (AMD) is the major cause of vision loss in old people over the age of 65 years. Notably, wet AMD is characterized by the formation of CNV, causing exudation, hemorrhage, retinal edema, pigment epithelial detachment, and fibrosis [24]. LncRNAs are shown the critical regulators of disease progression. They can regulate multiple signaling pathways or multiple biological processes, making them as the promising targets for disease treatment [25]. In this study, we revealed that IPW was significantly up-regulated in choroid tissues following laser-induced photocoagulation and in RF/6A cells following hypoxic stress. Silencing of IPW inhibited CNV formation *in vivo* and choroidal sprouting *ex vivo*, suggesting that IPW is a promising therapeutic target for neovascular ocular diseases.

Anti-VEGF therapy is the mainstream method for the treatment of neovascular ocular diseases [10]. However, a part of patients has no vision improvement after treatment and no respond to anti-VEGF treatment [26, 27]. The multifactorial features of CNV calls for the combinational drugs for efficient treatment. IPW is obviously induced in the formation of CNV. Silencing of IPW can inhibit choroidal neovascularization in laser-induced CNV model and choroidal sprouting. Moreover, IPW silencing mimics the effects of anti-VEGF drug on CNV formation. Hypoxia is a critical driver of CNV formation, which is tightly associated with the over-expression of VEGF and HIF-1α [28]. These factors act as transcription regulators involved in angiogenesis, erythropoiesis, and glucose metabolism to combat against hypoxic stress [29]. Notably, IPW silencing plus anti-VEGF drug could enhance the inhibitory effects on CNV formation compared with IPW silencing or anti-VEGF drug alone. IPW may be involved in the regulation of CNV formation through a novel pathway, which is different from VEGF signaling. Combined therapy may provide an appealing method for the treatment of CNV.

Gene imprinting plays a crucial role in early embryogenesis and later development [30]. IPW is identified as a long non-coding RNA in PWS locus. It is also shown as a critical regulator of DLK1-DIO3 region. IPW overexpression leads to the downregulation of maternally expressed genes (MEGs) in the imprinted DLK1-DIO3 locus, suggesting a role in gene imprinting regulation [21]. Reactivation of silent embryonic genes or aberrant imprinting may lead to the occurrence of human disease [31–33]. LncRNAs can regulate gene expression both in cis and in trans through the association with chromatin modifiers. By establishing genomic imprinting of target genes, lncRNAs are involved in several biological processes, including embryonic growth, pluripotency maintenance, cell differentiation, and neural-related functions. In this study, silencing of IPW lead sto reduced expression of miR-370. RNA pull-down assays confirmed the direct interaction between miR-370 and IPW. IPW silencing could lead to increased expression of miR-370. miR-370 shows an opposite expression pattern compared with IPW expression in laser-induced CNV lesions and endothelial cells following hypoxic stress.

Accumulating evidence reveals that miRNAs participate in regulating vascular endothelial function, vascular integrity and vasculogenesis [34–36]. For example, miR-221 and miR-222 can inhibit the proliferation and migration of vascular endothelial cells [37]. Inhibition of miR-23 and miR-27 can block angiogenesis *in vitro* and induce retinal development disorder. miR-23 and miR-27 are correlated to pathological vasculogenesis in laser-induced CNV model [38]. miR-370 inhibits the angiogenic activity of endothelial cells by targeting smoothened (SMO) and bone morphogenetic protein (BMP)-2 [39]. miR-370 also inhibits vascular inflammation and oxidative stress triggered by oxidized low-density lipoprotein through targeting TLR4 [40]. In this study, miR-370 is identified as a regulator of endothelial angiogenic function and CNV formation. miR-370 mimic significantly reduces the formation of CNV. IPW overexpression could rescue the inhibitory effects of miR-370 mimic on CNV formation *in vivo*. In an *ex vivo* choroidal sprouting assay, miR-370 mimic leads to reduced sprouting capacity of choroidal explants. By contrast, overexpression of IPW abolishes this effect and leads to cumulative outgrowth. Thus, it is not surprising that IPW/miR-370 interaction is involved in the CNV formation.

Taken together, PW is involved in the epigenetic regulation of CNV formation. Silencing of IPW reduces endothelial angiogenic effects *in vitro* and decreases CNV formation in laser-induced CNV model and *ex vivo* choroidal sprouting model. This study unveils a novel mechanism underlying CNV formation. Silencing of IPW is a promising method for the treatment of neovascular ocular diseases.

## MATERIALS AND METHODS

### Animal experiment

Animal experiments were performed in accordance with the ARVO Statement for the Use of Animals in Ophthalmic and Vision Research. The experiments were reviewed and approved by the Animal Care and Use Committee guidelines of Nanjing Medical University. C57BL/6 J male mice (6–8 weeks old) were chosen for this research. The animals were housed on a 12-hour light/dark cycle and fed a standard rodent chow.

### Intravitreal injection

After the photocoagulation, intravitreal injections were carried out under an operating microscope (66 Vision Tech, China). The vitrectomy ports were punctured approximately 2 mm posterior to the limbus using a 30-gauge blunt needle. The 33-gauge needle attached to a Hamilton micro syringe was carefully inserted through the pilot holes. Adeno-associated viral vector (AAV) or corresponding vehicle was subsequently injected into the vitreous cavity. The needle was held in the center of the vitreous cavity for 15–20 seconds to allow for intraocular pressure equilibration. After intravitreal injection, the ophthalmic antibiotic ointments (Sinqi, China) were applied onto the corneas to prevent infection.

### Choroid flat-mount isolectin-B4 staining

The mice were euthanized after photocoagulation. RPE complexes were enucleated and fixed with 4% paraformaldehyde (PFA; BL539A, Biosharp Biotechnology, China) for 1 h at room temperature. After washing, the choroids were cut into four quadrants, mounted onto glass slides, blocked with 1% bovine serum albumin (BSA) and 0.5% TritonX-100 for 30 min at 37°C. Then, the choroidal flat mounts were rinsed with PBS, incubated with Isolectin-B4 (1:100; IB4; L2895, Sigma-Aldrich, USA) overnight at 4°C, and observed under a fluorescent microscope (Olympus, Japan).

### Laser-induced CNV model

CNV was induced by laser photocoagulation with the rupture of Bruch's membrane as previously described [41]. Briefly, C57BL/6 J male mice (6–8 weeks old) were anesthetized by the intraperitoneal injection of ketamine (30 mg/kg; Pfizer, USA) and xylazine (5 mg/kg; Bayer, Germany), and both pupils were dilated by the topical administration of tropicamide (0.5%; Alcon, USA). Bruch’s membrane was ruptured at the 3, 6, 9, and 12 o’clock positions (532-nm wavelength; 50 μm spot size, 70 ms duration; 140 mW power) using the OcuLight GLx Laser System (Iridex, USA). The laser spots were performed 2 to 3 optic disk diameters away from the optic nerve head. The sign of air bubble at the site of photocoagulation indicated the disruption of Bruch’s membrane. Laser injury without hemorrhage was included in the study.

### Hematoxylin and eosin (HE) staining

The eyes were enucleated and the anterior segments were removed. The eyecups were fixed in formaldehyde-acetic acid-ethanol (FAA) fixative solution for 2 h at room temperature and transferred to 4% PFA at 4°C overnight. The tissues were dehydrated with the gradient ethanol (1 h each in, successively, 2 × 70% ethanol, 2 × 96% ethanol, and 3 × 100% ethanol) in a tissue dehydration automat (Zonway, China), incubated with xylene for 30 min and then embedded in paraffin. The eyes were sectioned into 6 μM thickness slices from the most prominent lesions using a microtome (Thermo Fisher Scientific, USA). The cross-section samples were deparaffinized for 15 min in a 65°C incubator. The slides were then rehydrated (2 min each in, successively, absolute ethanol, 90% ethanol, 80% ethanol, 70% ethanol, and distilled water). The sections were stained for 5 min with hematoxylin, rinsed with the distilled water for 1 min, counterstained with 1% hydrochloric ethanol for 30 sec, and washed with tap water for 1 min. The slides were then stained with eosin for 2 min and washed with tap water for 1 min afterward. After 2-min hyalinization in two baths of xylene, the sections were mounted with neutral resin. The tissue samples were observed and photographed. The lesion area and maximum thickness were quantified using Image J software. The image file was opened in Image J software. The tool bar was used to surround CNV neovascular area on the image with a freehand shape. A straight line perpendicular to RPE was created to measure the maximum thickness of CNV lesion. The areas and lines were analyzed after selection.

### Cell culture and transfection

RF/6A cells were cultured in Dulbecco’s Modified Eagle Medium (DMEM; 8120034, Gibco, USA) supplemented with 10% fetal bovine serum (FBS; 16140071, Gibco, USA) and 1% penicillin-streptomycin (15140122, Gibco, USA) in an atmosphere of 5% CO_2_ at 37°C. The cells at passages 5–8 were used. RF/6A cells were transfected with lipofectamine 3000 reagent (L3000015, Invitrogen, USA) at 50–60% confluence according to the manufacturer’s protocols. About 4 μL of lipofectamine 3000 were added to 200 μL DMEM and incubated for 5 min at room temperature. 200 μL of DMEM were mixed with 4 μg of siRNAs. Then, these solutions were mixed and incubated for 20 min at room temperature before the mixed solution were added to each well. After 6 h of incubation, the medium was changed to the complete basal medium and cultured for 24 h.

### RNA pull down assay

RF/6A cells were seeded on the 10-cm plate for 24 h. Next, these cells were transfected with biotin-labeled miR-370, or biotin-labeled miR-335 at a final concentration of 100 nM. They were coated with 10 μL per sample yeast tRNA (10 mg/mL stock; AM7119, Invitrogen, USA) and 10 μL BSA (10 ng/mL stock) and incubated in the lysis buffer (500 μL) under rotation at 4°C for 3 h. The beads were rinsed with wash buffer, and the sample lysates (600 μL) were mixed with pre-coated beads (50 μL per sample) and incubated at 4°C for 4 h on a rotator. The beads were then pelleted down to remove unbound materials at 4°C for 2 min at 500g and washed 6 times with 1 mL of wash buffer II (lysis buffer supplemented with 0.1% (v/v) Triton X-100) and 1 mL lysis buffer. IPW levels in the pull down samples were detected by qRT-PCRs.

### Choroidal sprouting assay *ex vivo*

After enucleation, RPE/choroid/sclera complex (choroid explants) from the peripheral area was dissected and cut into approximately 1 mm × 1 mm pieces. The choroid explants were immediately embedded in 30 μL growth factor reduced Matrigel (354230, Corning, USA) in 24-well tissue culture plates. The explants were grown in DMEM supplemented with 10% FBS and 1% penicillin-streptomycin at 37°C with 5% CO_2_. The sprouting area was quantified using Image J under 4 × magnification.

### Plasmid transfection

For overexpression, IPW cDNA was chemically synthesized and inserted into the pcDNA3.0 expression vector to generate pcDNA3.0-IPW. Empty-vector was served as a negative control. The plasmids were transfected into RF/6A cells using lipofectamine 3000 according to the manufacturer’s instructions. The expression of plasmids in RF/6A cells were confirmed by qRT-PCRs.

### RNA isolation and qRT-PCR

Choroidal tissue or RF/6A cells were lysed using the TRIzol reagent (15596026, Invitrogen, USA) following the manufacturer’s protocols. The total RNAs were treated with RNase free DNase (RNeasy Micro Kit; 74004, Qiagen, Germany) and equal amounts of RNA were reversely transcribed using the PrimeScript RT reagent Kit (RR037A, TaKaRa Biotechnology). cDNAs were synthesized using the random hexamers. qRT-PCRs were performed using the specific primers and SYBR green Master Mix (A25741, Thermo Fisher Scientific, USA) in the PikoReal Real-Time PCR System (Thermo Fisher Scientific). The qRT-PCR cycling conditions were 95°C for 5 min followed by 40 cycles of 95°C for 5 sec and 60°C for 30 sec. Relative gene expression was determined by the 2^-ΔΔCt^ methods. All qRT-PCR reactions were performed in triplicate and averaged.

### Cell proliferation assay

Cell proliferation was determined using the 5-Ethynyl-2'-deoxyuridine (EdU) incorporation assay (C0071S, Beyotime Biotechnology, China). Briefly, 1.0 × 10^5^ RF/6As were seeded in a 24-well cell culture plate. At 24 h post-transfection, RF/6A cells were fixed with 4% PFA for 15 min, incubated with 3% BSA for 5 min, permeabilized with 3% Triton X-100 for 15 min at room temperature, and incubated with the Click reaction cocktail for 30 min. Finally, RF/6A cells were stained with Hoechst 33342 to label cell nuclei.

### Cell viability assay

Cell viability was detected using 3-(4, 5-dimethylthiazol-2-yl)-2, 5-diphenyl-tetrazolium bromide (MTT) assay. About 1 × 10^4^ RF/6A cells were plated in 96-well culture plate and allowed to attach overnight. At 24 h after transfection, they were treated with 200 μmol/L CoCl_2_ for 24 h and incubated with 10 μL MTT solution (5 mg/mL; 1334GR001, Biofroxx, Germany) at 37°C for 3 h. The supernatant was discarded and the formazan crystals were dissolved in 100 μL isopropanol. The absorbance was determined at 570 nm by a microplate reader (Molecular Devices, USA).

### Cell apoptosis assay

The apoptosis of RF/6A cells was determined using propidium iodide/Calcein-acetoxymethyl (PI/Calcein-AM) staining according to the manufacturer's instruction. After the required treatment, RF/6A cells were stained with Calcein-AM (2 μg/mL; 22002, AAT Bioquest, USA) and PI (1 μg/mL; 1246MG100, Biofroxx, Germany) at 37°C for 15 min. The fluorescence images were obtained by a fluorescence microscope (Olympus, Japan). The percentage of PI-positive cells was counted using the Image J software.

### Tube formation assay

A total of 50 μL of ice-cold Matrigel Matrix (356234, Corning, USA) was coated on each well of the 24-well plates. The matrix was polymerized at 37°C for at least 45 min. About 2 × 10^5^ RF/6A cells were seeded onto the solidified matrix gel for 6 h. The pictures were taken under a light microscope. Tube formation ability was analyzed by calculating the total tube length (length of the capillaries) using the Image J software.

### Transwell migration assay

Transwell chamber (8.0-μm pore size; A190038, Millipore, USA) was used to conduct the migration assay. Briefly, 1 × 10^5^ RF/6A cells per well were seeded in the top chamber in 100 μL of serum-free medium. Then, 500 μL of complete medium was added to the bottom chamber as a chemoattractant. After migration for 12 h at 37°C, these non-invaded cells were removed by cotton swabs. The migrated cells were fixed with 4% PFA and stained with 0.1% crystal violet solution at room temperature for 30 min. The cells that passed through the filter were counted in 4 randomly selected fields.

### Statistical analysis

All continuous data were presented as mean ± SD with the indicated number (*n*) of independent experiments. Statistical significance was determined using Student’s *t*-test for two groups and analysis of variance (ANOVA) followed by the post hoc Bonferroni test for multiple groups if the data were normally and equally distributed. The nonparametric Mann-Whitney *U* test (2-group comparisons) or Kruskal-Wallis test followed by the post hoc Bonferroni test (multi-group comparisons) was used to compare abnormal distribution data. *P* < 0.05 were considered statistically significant. SPSS Statistics Base version 23.0 was used to analyze data distribution and differences between groups. GraphPad Prism 8 were used to export the bar graph.

## Supplementary Material

Supplementary Figures
