# Supplementary material for: Suppression of choroidal neovascularization by silencing of long non-coding RNA IPW
Source: Aging (Albany NY). 2021 Apr 4;13(7):10584–602. doi: 10.18632/aging.202822 (PMC8064148; doi:10.18632/aging.202822)
Supplement: Supplementary Figures [file aging-13-202822-s001.pdf]

SUPPLEMENTARY FIGURES

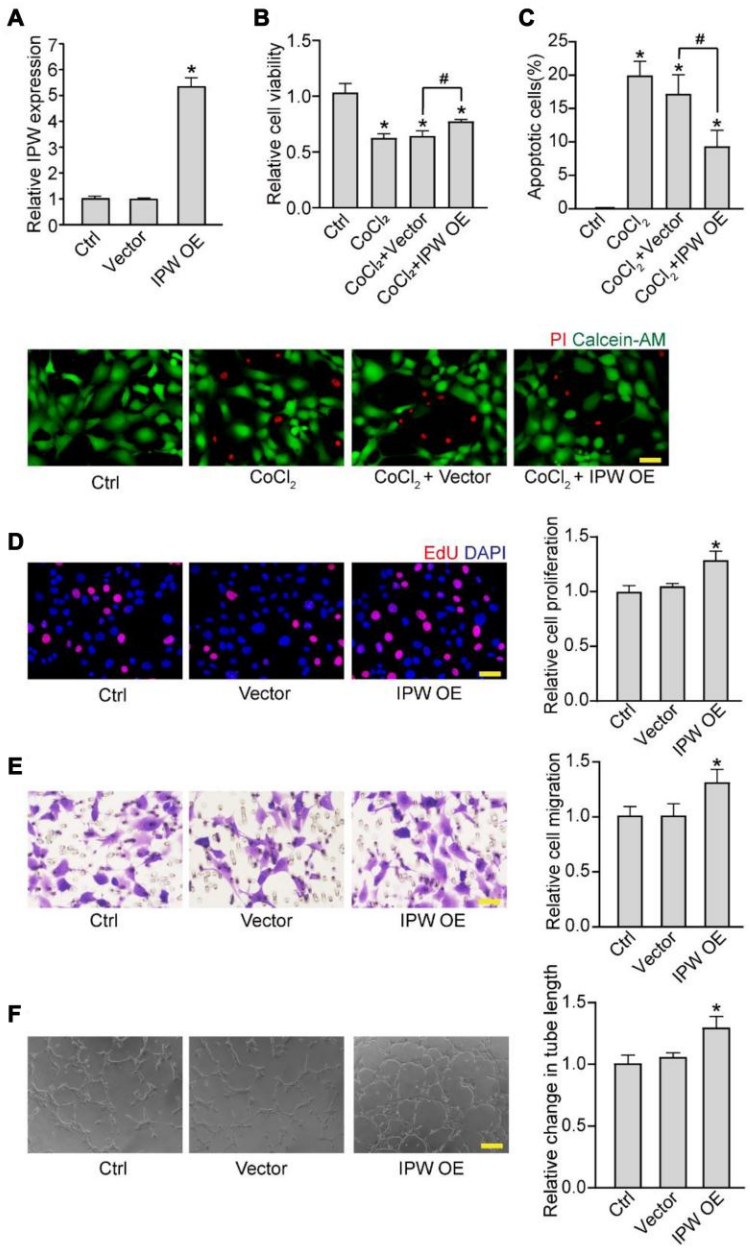

Supplementary Figure 1. lncRNA-IPW overexpression increases endothelial angiogenic function *in vitro*. (**A**) RF/6A cells were transfected with pcDNA3.0 vector (Vector), pcDNA3.0-IPW (IPW OE), or left untreated (Ctrl) for 24 h. qRT-PCRs were performed to detect IPW expression (n = 4; $^{*}P < 0.05$ versus Ctrl group; One-way ANOVA). (**B** and **C**) RF/6A cells were transfected with pcDNA3.0 vector (Vector), pcDNA3.0-IPW (IPW OE), or left untreated for 24 h, and then exposed with $CoCl_2$ (200 µmol/L) to mimic hypoxic stress for 24 h. The group without $CoCl_2$ treatment was taken as the Ctrl group. Cell viability was detected by MTT assays (B; n = 4; $^{*}P$ < 0.05 versus Ctrl group; $^{\#}P$ < 0.05 $CoCl_2$ + OE group versus $CoCl_2$ + Vector group; One-way ANOVA). Apoptotic cells were detected by PI/Calcein-AM staining. Green: live cells; red: dead or dying cells. Scale bar: 50 µm (C; n = 4; $^{*}P$ < 0.05 versus Ctrl group; $^{\#}P$ < 0.05 $CoCl_2$ + OE group versus $CoCl_2$ + Vector group; One-way ANOVA). (**D**–**F**) RF/6A cells were transfected with pcDNA3.0 vector (Vector), pcDNA3.0-IPW (IPW OE), or left untreated (Ctrl) for 24 h. Cell proliferation was determined by EdU incorporation assay. Blue: DAPI; red: EdU. Scale bar: 20 µm (D, n = 4; $^{*}P$ < 0.05 versus Ctrl group; One-way ANOVA). The migration of RF/6A cells was determined using Transwell assays. Scale bar: 50 µm (E, n = 4; $^{*}P$ < 0.05 versus Ctrl group; One-way ANOVA). The tube-like structures were observed 6 h after cell-seeding on the matrix. The average length of tube formation for each field was statistically analyzed. Scale bar: 200 µm (F, n = 4; *P < 0.05 versus Ctrl group; One-way ANOVA).

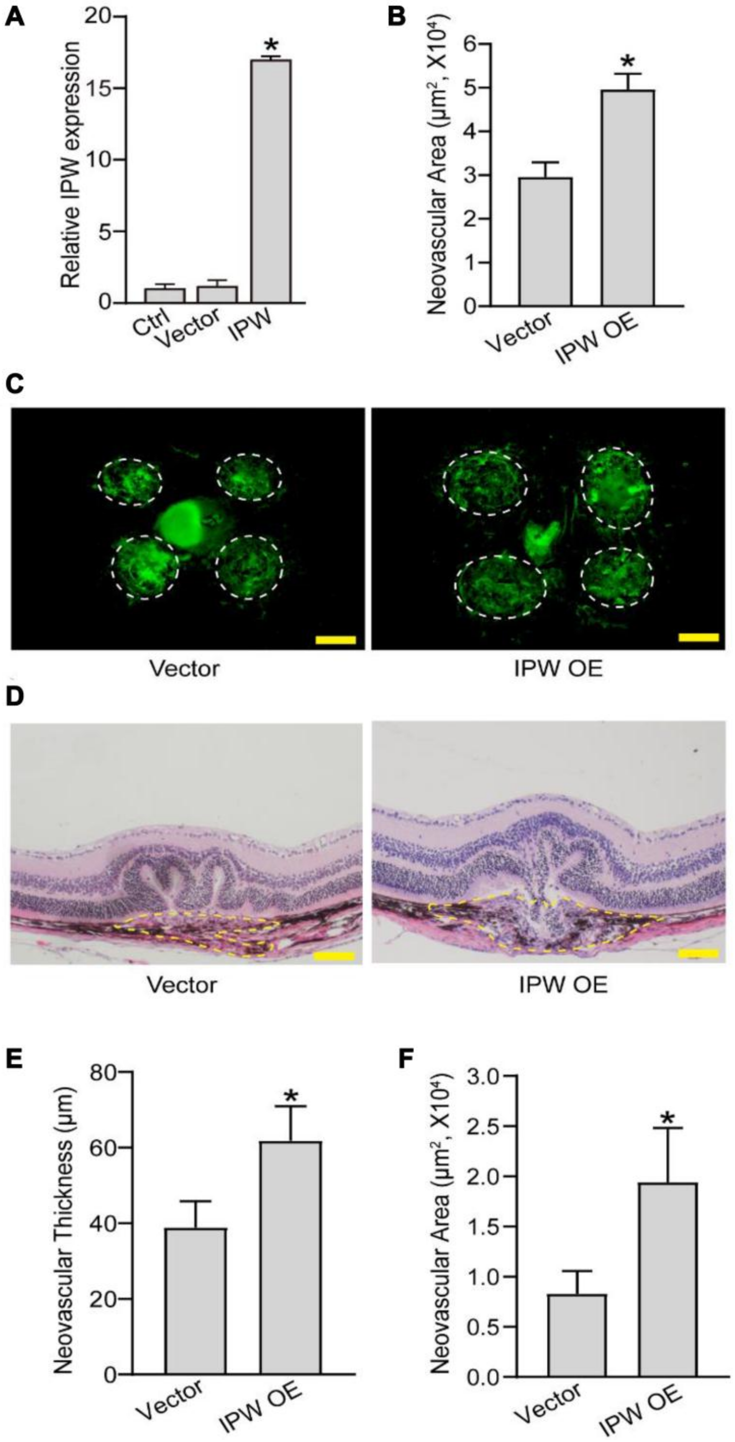

Supplementary Figure 2. lncRNA-IPW overexpression aggravates experimental choroidal neovascularization *in vivo*. (**A**) C57BL/6 mice received an intravitreal injection of AAV vector, IPW overexpression-AAV (IPW OE), or left untreated (Ctrl). qRT-PCRs were performed to detect IPW expression at day 14 after intravitreal injection (n = 5 animals/group; Kruskal-Wallis test; $^{*}P < 0.05$ versus Ctrl group). (**B**–**F**) C57BL/6 mice received an intravitreal injection of AAV vector or IPW overexpression-AAV (IPW OE). At day 14 after laser photocoagulation, the mice were euthanized and RPE/choroid complexes were dissected and flat-mounted. The blood vessels were labeled with Isolectin-B4. Neovascular area was quantified (B, n = 6 animals/group; Mann-Whitney U test). The representative images of Isolectin-B4-labeled flat-mounted choroid on day 14 after laser induction. Dashed lines delineate the lesion area. Scale bar: 200 μm (C). (**D**–**F**) Histological sections of HE-stained retinal sections from mice on day 14 after laser photocoagulation (n = 6 animals/group; Mann-Whitney U test). (**D**) Typical sections of laser injured eye stained with HE with the lesion delineated by the dashed line. Neovascular reactions were quantified by lesion thickness (**E**) and area (**F**). Scale bar: 100 μm.

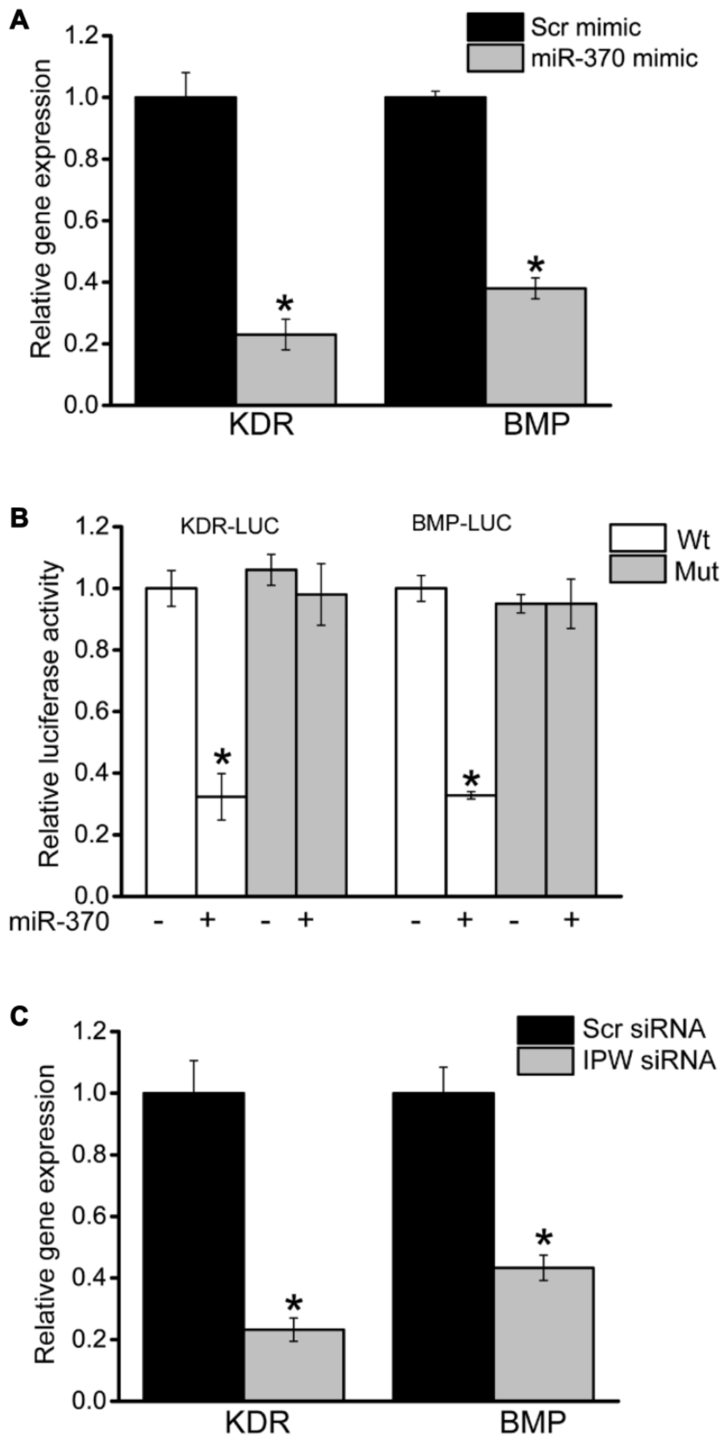

Supplementary Figure 3: Verification of the direct interaction of IPW-miR-370-KDR/BMP. (**A**) RF/6A cells were transfected with scramble (Scr) mimic or miR-370 mimic. Twenty-four hours after transfection, qRT-PCRs were performed to detect KDR and BMP expression (n = 4; $^*P < 0.05$; Student's t-test). (**B**) RF/6A cells were co-transfected wild-type (Wt) and mutant (Mut) LUC-KDR or LUC-BMP with or without miR-370 mimics. Luciferase activity was detected using the dual luciferase assay 48 h after transfection (n = 4; $^*P < 0.05$ Wt group versus Mut group; One-way ANOVA). (**C**) RF/6A cells were transfected with scramble (Scr) siRNA or IPW siRNA. Twenty-four hours after transfection, qRT-PCRs were performed to detect KDR and BMP expression (n = 4; $^*P < 0.05$; Student's t-test).
